# Whole genome sequence comparison of avian pathogenic *Escherichia coli* from acute and chronic salpingitis of egg laying hens

**DOI:** 10.1186/s12917-020-02369-5

**Published:** 2020-05-20

**Authors:** Louise Ladefoged Poulsen, Egle Kudirkiene, Steffen Lynge Jørgensen, Steven Philip Djordjevic, Max Laurence Cummins, Jens Peter Christensen, Henrik Christensen, Magne Bisgaard, Ida Thøfner

**Affiliations:** 1 Department of Veterinary and Animal Sciences, Faculty of Health and Medical Sciences, University of Copenhagen, Frederiksberg, Denmark; 2 The ithree institute, University of Technology SydneyUltimo, NSW, Australia; 3 Danish Technological institute, Taastrup, Denmark; 4 Viby Sjaelland, Denmark

**Keywords:** Salpingitis, *Escherichia coli*, Acute, Chronic, Whole genome sequencing, APEC

## Abstract

**Background:**

Infection in the oviduct (salpingitis) is the most common bacterial infection in egg laying hens and is mainly caused by *Escherichia coli*. The disease is responsible for decreased animal welfare, considerable economic loss as well as a risk of horizontal and vertical transmission of pathogenic *E. coli*. The outcome of salpingitis may be either acute or chronic. It has not yet been clarified whether the pathological manifestation is a result of the characteristics of the *E. coli* or whether the manifestation is associated with host factors such as host immunity.

**Results:**

From the core- and accessory genome analysis and comparison of 62 *E. coli* no genetic markers were found to be associated to either acute or chronic infection. Twenty of the 62 genomes harboured at least one antimicrobial resistance gene with resistance against sulfonamides being the most common. The increased serum survival and iron chelating genes *iss* and *iroN* were highly prevalent in genomes from both acute and chronic salpingitis.

**Conclusion:**

Our analysis revealed that no genetic markers could differentiate the *E. coli* isolated from acute versus chronic salpingitis in egg laying hens. The difference in pathological outcome may be related to other factors such as immunological status, genetics and health of the host. These data indicate that salpingitis is another manifestation of colibacillosis.

## Background

Avian pathogenic *Escherichia coli* (APEC) are considered as the most common bacterial infection in poultry [1]. Manifestations of colibacillosis in avian species are diverse and are often differentiated by the main anatomical location of the lesions and/or the proposed pathogenesis. The manifestations in poultry are usually extra-intestinal and most commonly occur in the respiratory organs (e.g. airsacculitis, polyserositis), the umbilicus (e.g. navel infections in newly hatched chicks, neonatal sepsis), the skin (e.g. “Swollen head syndrome”, cellulitis) and the reproductive organs [e.g. salpingitis, oophoritis, peritonitis]) [1]. All manifestations can be associated/complicated with a septicemic phase often with an acute and/or fatal outcome. In egg laying hens infections of the reproductive tract (e.g. salpingitis with or without concurrent peritonitis) with *E. coli* is considered the most common bacterial infection [1–4] and therefore of major concern both economically [5] and in regard to animal welfare. Salpingitis in breeder animals furthermore poses a risk for infecting offspring through the vertical transmission of *E. coli* [6]. In particular, *E. coli* transmitted to offspring via the egg [6] cause economic and welfare problems to broiler production since one infected hen may pass the bacteria to a large number of offspring, which then can pass it on horizontally to flock mates. Hens suffering from chronic infections may continue to be in lay for an unknown period despite the infection [7], thereby contributing to the spread of pathogenic *E. coli.* Based on pathology, salpingitis may be divided into acute and chronic infections.

The different outcomes of salpingitis may be due to host factors, different virulence characteristics of the pathogens, or both [8]. The severity and outcome of *E. coli* infections in the reproductive tract are highly dependent on the strain involved in the infection, and strains originating from other manifestations of colibacillosis may also have potential to cause lesions in the reproductive tract of egg laying hens [8]. For other bacterial diseases in poultry, it has been described that certain subpopulations are associated to certain disease outcomes. Amyloid arthropathy is a disease which is caused mainly by *Enterococcus faecalis,* an opportunistic pathogen like *E. coli* [9]. Investigations of the aetiology of the diseases revealed that in particular ST 82 of *E. faecalis* [10] is associated to the chronic infections, which then give rise to the deposition of the amyloid fibril proteins in the joints of the affected birds.

It has recently been proposed, that certain APEC strains serve as primary pathogens and that these may have an overlap in genetic traits (e.g. virulence associated genes (VAG), plasmids, and antimicrobial resistance genes) from the pool of human-associated extra-intestinal pathogenic *E. coli* (ExPEC), thus also posing a risk to public health [11]. Comparative genomics has demonstrated that APEC and ExPEC share a wide range of genomic traits [12, 13]. This is underlined by the ability of UTI89 (ST95), a common UPEC, to cause disease in an experimental avian salpingitis model to a similar severity as for a highly virulent APEC (ST117) [14], thus emphasizing the zooanthroponotic/anthropozoonotic potential of *E. coli.* Furthermore, there is also an overlap in among *E. coli* sequence types (ST’s) giving rise to disease in both broiler chickens and adult birds [6, 15].

Genomic identification of subpopulations of *E. coli* related to different outcomes of salpingitis may be a useful tool used for future control of *E. coli* infections in egg laying hens of both broiler and layer production systems. If subpopulations of *E. coli* can be associated with chronic salpingitis this would be of great economic and welfare importance in broiler production due to the possibility of interventions to prevent vertical transmission of *E. coli* from broiler breeders to broilers. Likewise, if subpopulations can be associated with acute salpingitis, an often-reported manifestation in *E. coli* outbreaks in egg laying hens, a risk assessment for handling the infection to limit the outbreak is possible. Furthermore, knowledge of certain APEC subpopulations associated with certain manifestations (e.g. salpingitis, airsacculitis etc.) and/or bird types may be valuable for design of vaccines against *E. coli.*

## Results

### The association of pathological manifestation with MLST type and serotypes

**Fig. 1:**
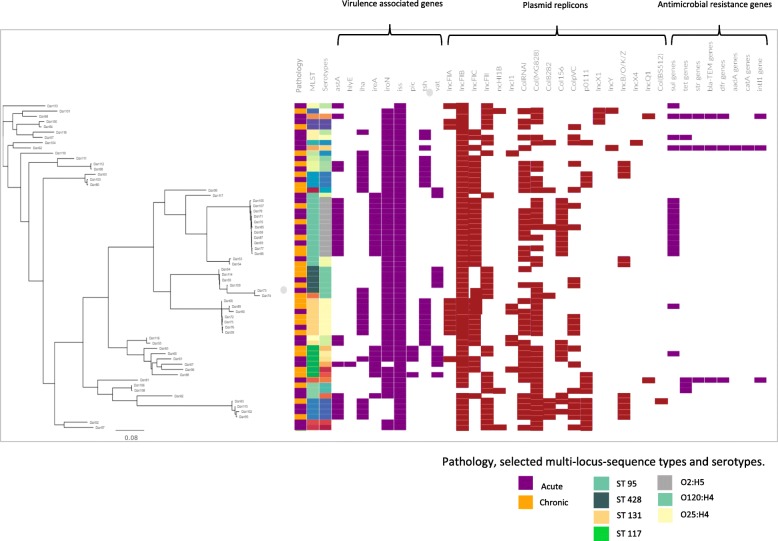
Distribution of virulence genes, plasmid replicons, resistance genes and the class 1 integron-integrase (intl1 gene, as a proxy for multidrug resistance) of 62 *E. coli* isolated from acute and chronic salpingitis of broiler breeder hens. The phylogenetic tree is based on core genome SNP analysis and the colour codes for the most prevalent multi-locus sequence types and serotypes are illustrated under the figure

Based on the 7 gene MLST scheme, a total of 22 different STs were identified (Table 1). ST 95 and 131 were the most prevalent STs constituting 34% of the samples in the present study. Additional analysis revealed no significant difference in the number of ST 95 isolated from acute or chronic salpingitis (Fischer’s exact test, *p* = 0.77). The same was true for ST 131 (*p* = 0.41). The raw read mapping of 62 sequenced *E. coli* isolates to a reference strain (*E. coli* K12 MG1655) resulted in a total of 83,216 single-nucleotide polymorphisms (SNPs). In Fig. 1, isolates clustered according to MLST - and serotypes and not according to the pathological status of the bird the *E. coli* originated from.

**Table 1:** Distribution of multi-locus sequence types (MLST) within 62 *E. coli* isolated from acute and chronic salpingitis

MLST	Acute salpingitis (n)	Chronic salpingitis (n)	Total isolates (n)
23	1	0	1
48	1	0	1
58	1	0	1
69	1	0	1
88	1	1	2
93	1	2	3
95	8	6	14
101	2	0	2
109	0	1	1
115	2	2	4
117	2	4	6
131	2	5	7
162	1	0	1
352	0	1	1
428	2	4	6
616	0	1	1
648	2	1	3
1146	1	0	1
1518	1	0	1
1841	1	1	2
3346	2	0	2
5628	1	0	1
Total	33	29	62

### Virulence associated genes

The presence of selected virulence associated genes (VAGs), important for establishment of an infection in the host [16, 17], revealed no association to the two different pathological outcomes (Fig. 1). For example, genes involved in the iron metabolism and serum survival were present in all strains, with *iss* and *iroN* being most prevalent*.* The presence / absence of VAGs was highly associated to ST and not pathological outcome (Fig. 1). The presence / absence of replicons were also found to be associated to ST rather than the outcome of salpingitis (Fig. 1).

### Resistance gene analysis

**Fig. 2:**
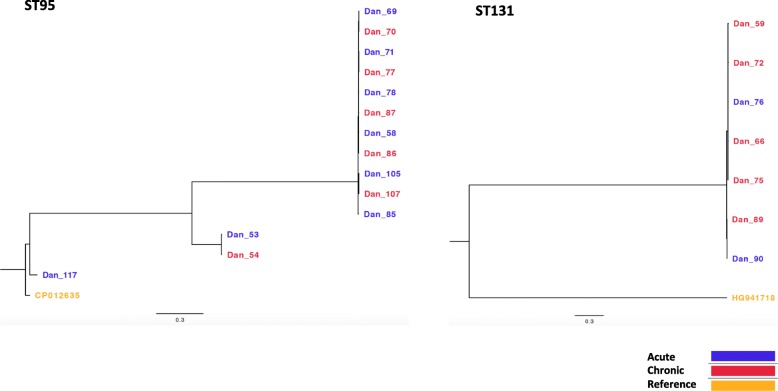
Phylogenetic trees based on core genome SNP analysis of the two most prevalent sequence types (ST). ST 95 and 131. *E. coli* CP012635 (ST95) and HG941718 (ST131), were used as reference for the phylogeny of ST95 and ST131, respectively

Twenty out of 62 genomes had at least one gene encoding for antimicrobial resistance (Fig. 1). No association between the resistance genes and the form of salpingitis was observed. The most frequently observed antibiotic resistance genes were *sul* genes (*sul1* and *sul2*), present in 17/62, of which 11 of 14 ST 95 strains harbored the *sul2* gene. The remaining three ST 95, which did not harbour the *sul2* gene formed two sub-cluster which is visualized in Fig. 2. Three unrelated strains harbored four or more resistance genes [18]. Three genomes out of the 62 genomes (Dan62, Dan68 and Dan81) also carried the class 1 integron-integrase gene (accession number: HQ730118.1) a reliable proxy for multiple drug resistance in enterobacteriaceae [18]. These three isolates carried four to seven resistance genes in contrast to the remaining isolates, of which 22% (13/59) carried only genes associated with sulfonamide resistance (*sul1*/*sul2*) and one isolate carried sulfonamide resistance and tetracycline resistance (*sul1*-like gene and *tet*(A)). Two isolates carried only tetracycline resistance (Fig. 1).

### Core SNP phylogeny and accessory gene analysis within the most prevalent MLST types

As no specific ST of *E. coli* was associated to the pathological status of the host, it was investigated whether any sub-clustering associated to the pathological status within the two most common STs was present. ST 95 and 131 were the most prevalent STs comprising 34% (*n* = 14) and 11.3% (*n* = 7), respectively. The core genome SNP analysis of 14 *E. coli* isolates belonging to ST 95 resulted in three sub-clusters, which differed with a maximum of 3138 SNPs. The small sub-clusters consisted of one isolate from acute salpingitis and the reference strain. The other sub-cluster consisted of isolates originating from both acute and chronic salpingitis (Dan53 and Dan54) differed with only six SNPs (Fig. 2). These two isolates originated from the same farm and were isolated three months apart. Within the large sub-cluster consisting of 11 isolates, three isolates (Dan58, Dan69 and Dan87) differed by 12 SNPs. These highly similar isolates originated from both acute and chronic salpingitis but from three different farms five months apart (Fig. 2). The phylogenetic tree of seven *E. coli* isolates of ST 131 all clustered together and differed with a maximum 69 SNPs.

**Fig. 3:**
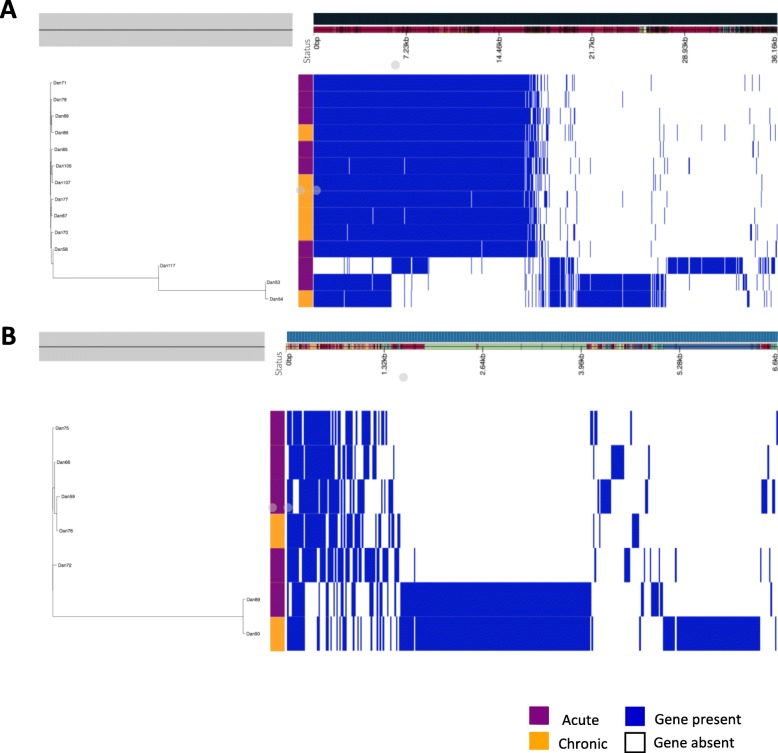
Hierarchic gene presence / absence tree (left) and accessory genome distribution (right) calculated from the pan-genome analysis of A) the isolates belonging to ST95 and B) the isolates belonging to ST131. In the status bar, isolates from the acute cases are shown in blue, and isolates from the chronic cases are shown in orange

In addition to the SNP analysis a hierarchic gene presence / absence tree was generated and accessory genome distribution was calculated from the pan-genome analysis to evaluate if any accessory genes may be associated to acute or chronic salpingitis. Figure 3 reveals that the presence / absence of genes was not associated to acute or chronic salpingitis either. We found that the clustering of isolates were independent of pathological status and the presence / absence of genes (Roary output). In addition, we found no statistically difference in the gene content between isolates from acute and chronic salpingitis. Scoary was applied for this statistical analysis.

## Discussion

The classification of pathogenic *E. coli* can be based on the pathotypes described by Kaper et al. [19] where *E. coli* is divided into Extra-intestinal pathogenic - and enteropathogenic *E. coli*. Each of the two pathotypes contains several subpathotypes based on the lesions and virulence factors associated to the *E. coli* (e.g. Avian pathogenic *E. coli* (APEC), Uropathogenic *E. coli* (UPEC) and Meningitis-associated *E. coli* (MNEC))*.* In theory, classification of APEC might be further differentiated into subgroups of subpathotypes of *E. coli* causing e.g. salpingitis, omphalitis/neonatal sepsis, swollen head syndrome and airsacculitis/polyserositis.

The possibility to predict the pathological outcome of salpingitis based on the genotype of *E. coli* would be of both economic and welfare importance in egg laying hens in the future. Broiler breeder hens carrying a chronic salpingitis may increase the risk of transmitting APEC to the offspring as well as increase the risk of horizontal transmission to flock mates due to a prolonged shedding time. Hens suffering from these chronic infections would be of particular interest to identify as they may carry the infection for weeks with no or few clinical symptoms, thus in practice difficult to identify by the personnel managing the birds. Pathogenic *E. coli* may be found in the intestine [20, 21] and can be transmitted to the offspring via the shell of the egg.

Genome analysis of 62 *E. coli* isolated from 33 acute cases of salpingitis and 29 chronic cases revealed that none of the investigated genetic traits of the *E. coli* could be associated to a specific manifestation of salpingitis. In 2011 Maturana et al. [22] described that avian pathogenic *E. coli* could be grouped into subpathotypes based on the association to specific diseases/manifestations; septicaemia, omphalitis and swollen head syndrome. Maturna did not go deeper into possible differentiation of the *E. coli* within each of the subpathotypes, which was the aim of the present study. To the best of our knowledge, a specific subgroup of subpathotype responsible for salpingitis has not yet been described. In the present study, 22 different STs were identified as the cause of salpingitis and no genetic traits were found to be associated to the pathological outcome of salpingitis. This observation indicates that salpingitis may be caused by a variety of different *E. coli.* Lilje et al. [23] investigated whether *Staphylococcus aureus* which cause only bloodstream infection could be distinguished from *S. aureus* isolates which cause infective endocarditis in addition to the bloodstream infection. However, it was concluded that the genotype of *S. aureus* cannot predict the outcome of the infection. The same conclusion must be drawn in this study; the genotype of *E. coli* cannot predict the pathological outcome of salpingitis in egg laying hens. An alternative hypothesis could be that host factors could be associated to the outcome of the disease. A recent study on experimentally induced *E. coli* salpingitis by [24] Olsen et al. demonstrated that strains, despite primarily associated to other typical manifestations (e.g. swollen head syndrome), were able to confer disease at a level similar to strains isolated from salpingitis. However, considerable host variation was observed between the birds receiving the same strain, no matter the origin of the strain.

The high abundance of ST 95 and ST 131 in this study is not unexpected. Both STs are commonly associated with disease and outbreaks in poultry where they may cause considerable economic loss and reduced animal welfare due to increased morbidity and mortality of the affected flocks [5, 25–27]. It may also be suggested that these STs have a zoonotic potential as they are also commonly found among human infections, the two STs are among the most common STs in bloodstream- and urinary tract infections [28–31].

The fact that both acute and chronic isolates were represented in the highly similar sub-clusters of ST 95 and ST 131 in the SNP analysis further indicates that the pathological manifestation may not be associated to genetic traits. This is in line with a recent study describing considerable host overlap (human and poultry) in ST 95 which finds equally low numbers of SNPs (30–57 SNPs) within sub-clusters of ST 95 [32]. This indicates that some genotypes of *E. coli* ST 95 are not host-specific.

Regarding presence of genes encoding for antimicrobial resistance the present study reveals a low number of isolates carrying more than four antimicrobial genes. Notably, the three isolates that did carry more than four genes also carried the class 1 integron-integrase gene. A recent study from Australia found that 48% of APEC isolates carried the class 1 integron-integrase gene [33] in comparison to 5% of the Danish isolates in the present study. In Denmark and Australia the use of antimicrobials in the poultry production is very restricted [34, 35]. The difference between the prevalence of the class 1 integron-integrase gene between the Danish and the Australian isolates could be explained by the fact that growth promoters were banned in Denmark from 2000 while in Australia growth promoters, like arsenicals, which were used until 2012 and in 2018 arsenicals were deregistered and taken of the market [36]. In addition, we found that a high concentration of arsenic also increased the abundance of a class 1 integron, an integrase-dependent system facilitating the horizontal transfer of genes conferring resistance to heavy metals and antibiotics [37]. While the majority (79%) of the ST 95 isolates carried resistance genes against sulfonamide in the current study this carriage rate was not mirrored in a study of ST 95 where only 16.1% of the 323 *E. coli* ST 95 isolates carried genes associated with sulfonamide resistance [32]. In the Danish Surveillance program of antimicrobial consumption and resistance (DANMAP) it is described that 17% of *E. coli* isolates from broilers in Denmark were resistant to sulfonamides in 2016 [38]. In this study 17/62 (27%) of the *E. coli* were resistant against sulfonamide. In 2015, poultry production in Denmark used 445 kg of active compound sulfonamide and trimethoprim [39] in comparison to 37 kg in 2018 [34]. The somewhat higher level of resistance against sulfonamides in the present study may reflect the fact that unusual high amounts of sulfonamides where used in poultry flocks during 2014 and 2015 when the isolates for this study were collected.

Poultry intestinal carriage of extra-intestinal pathogenic *E. coli* (ExPEC) which causes extra-intestinal disease in humans may be of epidemiological relevance and concern as the STs found are similar to the most prevalent STs from this study [40–43]. *E. coli* has been identified in both eggs and poultry meat [28, 31, 44, 45] which may serve as a vector for *E. coli* Moreover it is suggested that certain lineages within ST131 have acquired traits which may favor intestinal colonization despite being characterized as ExPEC [46].

This study is limited by the fact that the sampling of *E. coli* from salpingitis included only four Danish flocks however, the authors believe that the flocks represent the situation in the other countries receiving breeders from the same grandparent flocks and having very high standards in biosecurity on farm [39]. A genomic comparison of *E. coli* isolated from salpingitis to *E. coli* isolated from other typical lesions caused by *E. coli* could reveal if subgroups of subpathotypes of *E. coli* are associated to the different lesions as described in humans. However, such investigations require a high level of detail on the metadata (e.g. bird type/species, age, organ isolated, pathological description, outbreak status, disease/no disease, mortality rates, as well as other epidemiological information), thus enabling high quality selection of genomes for further genomic studies on publically available genomes. At present, metadata associated to available APEC genomes may often be limited to bird type and/or assigned as colibacillosis without other information or description of the actual lesions, clinical course of infection or epidemiological data. This lack of information hampers global comparison of which subgroups of APEC that could be associated to certain host types/manifestations and hereby provide valuable information for development of control strategies of APEC.

## Conclusions

In this investigation, it was found that sub-populations of *E. coli* are not associated to the pathological outcome of salpingitis. The pathological outcome of salpingitis may be related to other factors such as the immunological status, genetics and health of the host.

## Methods

### Aims and design of the study

In this study, we aim to investigate whether specific subpopulations of *E. coli* are associated with the two different pathological outcomes of salpingitis (acute or chronic), and secondly if certain ST’s or VAGs may be associated to a specific outcome of the disease.

Four Danish broiler breeder farms (Ross 308) were followed for a full production period (20–60 weeks) in 2014–2015. Dead broiler breeders were collected daily for post mortem and bacteriological examination. Dead birds were stored at − 20 °C immediately after collection and until post mortem examination [6]. In total, 997 broiler breeders were subjected to post mortem examination.

### Post mortem examination

All birds underwent a full post mortem examination including bacteriological sampling when vascular disturbances, discoloration, exudations and/or enlargement of liver or spleen were present as signs of infection in the oviduct.

### Definition of pathological manifestations

Salpingitis lesions were divided into acute or chronic. The acute lesions were characterized by vascular disturbances (e.g. hyperemia, capillary dilation, and congestion) in the ovaries and oviduct. Presence of fibrin and pus in the oviduct were commonly found, and sometimes accompanied with fibrinous exudation in the peritoneum or around the follicles in the ovary. The hens were either in lay (fully active ovarian follicles) or in the process of going out of lay (onset of follicular regression of the large ovulatory follicles). Diffuse necrosis of the liver was commonly observed. Whereas chronic salpingitis was characterized by various amounts of caseous exudate in the oviduct. Birds most often presented with considerable follicular regression with small follicles (< 10 mm) or an atrophied ovary, and were thus out of lay at the time of death [47, 48]. In such cases, no signs of a systemic infection were observed. Furthermore, the majority of the birds also displayed lesions like nephropathy and emaciation or cachexia, which may have contributed to the death of the bird.

### Bacteriology

Bacteriological samples were collected during post mortem examination with a sterile cotton swab after sterilizing the surface of the salpinx and liver with a hot iron. Samples from salpinx and liver were immediately plated on blood agar plates (BA) prepared with 5% calf blood in a blood agar base (Oxoid, Basingstoke, UK). All samples were incubated aerobically overnight at 37 °C. From plates showing dense growth of presumptive *E. coli* colonies (medium size, circular, low convex with a light greyish colour) in pure culture one single colony were sub-cultured in Brain-Heart-Infusion broth (Difco, Brøndby, Denmark) before adding glycerol to 15% for storage at − 80 °C until further characterization. Species verification prior to pulsed-field-gel-electrophoresis (PFGE) analysis was done on a subpopulation of isolates by MALDI-TOF MS [49].

### Pulsed-field-gel-electrophoresis (PFGE)

*Escherichia coli* isolates (*n* = 178) were typed by pulsed-field-gel-electrophoresis (PFGE). Non-clonal isolates were selected for whole genome sequencing (WGS). PFGE was performed as previously described [6].

### Selection of *E. coli* for whole genome sequencing

From each of the four farms a dendrogram based on Dice similarity (1% similarity and 1% optimization) was constructed (GelCompar II, Applied Math, Sint-Martens-Latem, Belgium). Clonal *E. coli* was defined as isolates with an identical PFGE pattern. From each farm one *E. coli* from acute and chronic episodes of salpingitis were selected, when present, from each cluster. Identical PFGE patterns were present across farms, meaning that more *E. coli* with identical PFGE type would be selected if they originated from different farms. In total, 33 *E. coli* isolates were selected from acute salpingitis and 29 *E. coli* from chronic salpingitis.

### Whole genome sequencing

The gDNA from each selected isolate was extracted from overnight cultures in LB medium using DNeasy blood and tissue kit (Qiagen, Ballerup, Denmark) kit, according to manufacturer’s instructions. The quality of DNA was assessed using gel electrophoresis, and the concentration using Qubit dsDNA HS Assay kit (Thermo Fisher Scientific, Scoresby, Australia). Whole genome sequencing libraries were prepared from separate aliquots of sample gDNA using the Illumina Nextera DNA kit with modifications.

The PCR-mediated adapter addition and library amplification was carried out using customized indexed i5 and i7 adaptor primers (IDT, Coralville, IA, USA), which were developed based on the standard Nextera XT Indexed i5 and i7 adapters (e.g. N701-N729 and S502-S522). Libraries were then pooled and size selected using SPRI-Select magnetic beads (Beckman Coulter, Lane Cove West, Australia). Finally, the pooled library was quality checked and quantified on an Agilent Bioanalyzer 2100 using the DNA HS kit (Agilent, Santa Clara, CA, USA). Whole genome sequencing was performed using an Illumina HiSeq 2500 v4 sequencer in rapid PE150 mode (Illumina, San Diego, CA, USA).

The genome sequences of *E. coli* has been deposited in NCBI under the bioproject ID PRJNA507325.

### Quality control, assembly and annotation of sequences

Sequence read quality was initially assessed using FastQC (v0.11.5) (http://www.bioinformatics.babraham.ac.uk/projects/fastqc/). Illumina raw reads passing quality control were assembled in EnteroBase (www.enterobase.warwick.ac.uk). Enterobase Assembly pipeline uses SPADes for a high-quality assembly of bacterial genomes https://enterobase.readthedocs.io/en/latest/pipelines/backend-pipeline-qassembly.html. N50 were between 78,400 and 466,670 and the numbers of contigs were between 72 and 230. Genomes were annotated in Prokka (v1.2) [50] and the sequence types, serotypes as well as the presence of virulence genes and plasmid replicons were retrieved from EnteroBase (http://enterobase.warwick.ac.uk/species/index/ecoli).

### Single nucleotide polymorphism analysis (SNPs)

A phylogenetic tree including all 62 genomes based on 83,216 base pairs mapped to *E. coli* K12 MG1655 (Fig. 1) was constructed by the use of CSI Phylogeny 1.4 [51] and visualized in phandango [52].

In addition, a SNP analysis of the two most prevalent STs were performed using CSI phylogeny tool. The two phylogenetic trees were constructed separately using *E. coli* ST95 CP012635 as a reference to compare all ST95 genomes, and *E. coli* HG941718 to compare all ST131 genomes. The phylogenetic trees from the CSI Phylogeny are presented as Maximum likelihood trees.

### Accessory genome analysis

Roary (v3.6.0) [53] was employed to determine the core and accessory genome of 62 *E. coli* and Scoary (v1.6.16) [54] to calculate Bonferroni statistical test of the association of genes in relation to the different clinical manifestations of salpingitis [54]. The presence / absence of virulence genes, plasmid replicons and antimicrobial genes in the accessory-genomes in relation to the clinical manifestation were visualized using Phandango [52] in Fig. 1.

## Data Availability

The genome sequences of the 62 *E. coli* are available in NCBI under the bioproject ID PRJNA507325.

## Abbreviations

APEC: Avian Pathogenic *E. coli*
ExPEC: Extra-intestinal pathogenic *E. coli*
MLST: Multi-locus sequence typing
SNPs: Single-nucleotide polymorphisms
ST: Sequence type
VAG: Virulence associated gene
